# Effects of Gaze Fixation on the Performance of a Motor Imagery-Based Brain-Computer Interface

**DOI:** 10.3389/fnhum.2021.773603

**Published:** 2022-01-24

**Authors:** Jianjun Meng, Zehan Wu, Songwei Li, Xiangyang Zhu

**Affiliations:** ^1^Department of Mechanical Engineering, Shanghai Jiao Tong University, Shanghai, China; ^2^Department of Neurosurgery, Huashan Hospital, Fudan University, Shanghai, China

**Keywords:** brain-computer interface (BCI), electroencephalography (EEG), motor imagery, gaze fixation, covert attention

## Abstract

Motor imagery-based brain-computer interfaces (BCIs) have been studied without controlling subjects’ gaze fixation position previously. The effect of gaze fixation and covert attention on the behavioral performance of BCI is still unknown. This study designed a gaze fixation controlled experiment. Subjects were required to conduct a secondary task of gaze fixation when performing the primary task of motor imagination. Subjects’ performance was analyzed according to the relationship between motor imagery target and the gaze fixation position, resulting in three BCI control conditions, i.e., congruent, incongruent, and center cross trials. A group of fourteen subjects was recruited. The average group performances of three different conditions did not show statistically significant differences in terms of BCI control accuracy, feedback duration, and trajectory length. Further analysis of gaze shift response time revealed a significantly shorter response time for congruent trials compared to incongruent trials. Meanwhile, the parietal occipital cortex also showed active neural activities for congruent and incongruent trials, and this was revealed by a contrast analysis of R-square values and lateralization index. However, the lateralization index computed from the parietal and occipital areas was not correlated with the BCI behavioral performance. Subjects’ BCI behavioral performance was not affected by the position of gaze fixation and covert attention. This indicated that motor imagery-based BCI could be used freely in robotic arm control without sacrificing performance.

## Introduction

Brain-computer interface (BCI) technology has attracted widespread attention in both research and clinical applications. It has opened doors to improving the life quality of patients who suffer from neurological disorders such as spinal cord injury and amyotrophic lateral sclerosis (Bouton et al., 2016; Soekadar et al., 2016; Chaudhary et al., 2017; Moses et al., 2021; Willett et al., 2021). Motor imagination (MI) utilizes a rehearsal of limb movement, and it is a commonly used strategy to build up a noninvasive BCI (Pfurtscheller and Neuper, 2001; He et al., 2020). In addition, MI-based BCI demonstrates promising applications of operating assistive devices such as a wheelchair (Long et al., 2012; Tonin et al., 2019) and a robotic arm (Meng et al., 2016; Edelman et al., 2019), rehabilitating stroke patients (Biasiucci et al., 2018), etc.

Motor imagination is an endogenous mental process; it provides a gaze-independent control way (Wolpaw et al., 2002). One evident example is that MI classification of the left hand and right hand in eyes closing could be comparable to the classification in the eyes opening scenario (Brandl et al., 2016). A typical MI paradigm with eyes opening requires the subjects to focus on the center of a center-cross during the control period. Feedback will prompt the decoded results at the end of each trial (Pfurtscheller and Neuper, 2001). Many previous studies take advantage of this conventional design (Van Gerven and Jensen, 2009; Brunner et al., 2011; Yao et al., 2013; Ang et al., 2014; Zhang et al., 2015). However, plenty of studies provide more feedback information, e.g., the dynamical process of the control is continuously available (Wolpaw and McFarland, 2004; Wander et al., 2013; Wei et al., 2013; Meng et al., 2016; Edelman et al., 2019). This dynamical control process is entirely meaningful to real applications such as operating a robotic arm or a wheelchair since subjects need to interact with the environment in real-time (Meng et al., 2016; Edelman et al., 2019).

In the above real applications, subjects might not have a visible center cross to focus on, but they still have the targets in their mind. In this sense, it is interesting to investigate the efficacy of gaze fixation during the MI-based BCI. For example, whether there is any performance difference between gaze fixation on the center cross and gaze fixation on the indicated target. Furthermore, there are additional neural activities in the process of robotic arm control, e.g., subjects have to covertly pay attention to the movements of the robotic arm if they choose to fix their gaze at the target or vice versa. These simultaneous multiple neural activities happen not only for static target reach-and-grasp (Meng et al., 2016) but also for continuous cursor tracking (Edelman et al., 2019). Therefore, it is also interesting to see the influence of covert attention on MI-based BCI’s performance.

Eye movement and gaze fixation points have been utilized as additional features for improving the performance of MI-based BCI (Frisoli et al., 2012; Cheng et al., 2020). However, to our knowledge, research of motor imagination while rigorously controlling the position of subjects’ gaze is scarce. In this study, gaze fixation is a secondary task compared to the primary MI tasks. First, we aim to investigate the effect of gaze fixation at different locations when performing motor imagery tasks. Second, we want to explore the neural activity induced by the secondary task and its influence on the primary tasks.

## Materials and Methods

Fourteen subjects (1 female; all are right-handed subjects; average age 22.9 ± 4.6 years; range, 20–38) were recruited in a single session of BCI online experiment with cursor control. One subject was an experienced BCI user, and another two subjects had several sessions of BCI practice when developing the BCI program. All the other subjects were naïve BCI subjects. Additionally, all of the subjects were naïve to the dual tasks before participating in this study. All procedures and protocols were approved by the Institutional Review Board of Shanghai Jiao Tong University. Written informed consents were obtained from all of the participants before they agreed to participate in the experiment.

### Experimental Setup

**FIGURE 1:**
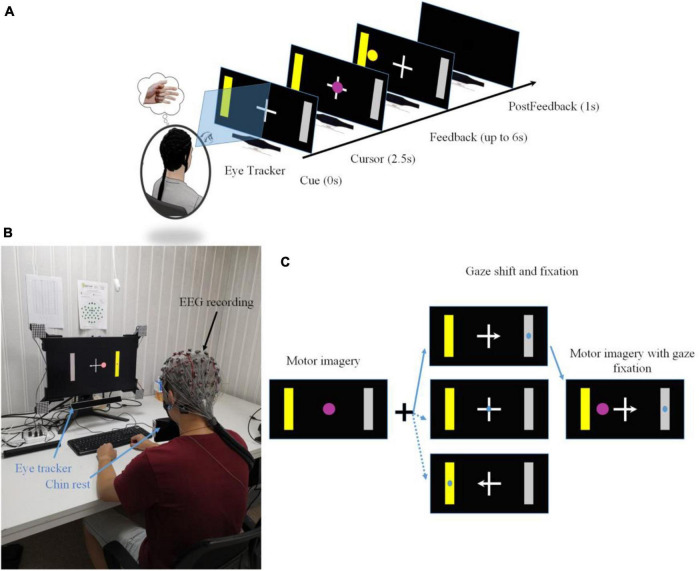
**(A)** A single trial structure of a motor imagery based BCI with gaze shift and fixation. **(B)** The experimental setup of motor imagery based BCI with eye tracking. A chin rest was used to secure the position of a subject’s head. **(C)** Three control conditions resulting from the congruence between a motor imagery task and a gaze shift and fixation position.

A 64 channels g.HIamp system (g.tec Medical Engineering, Austria) and suitable size g.GAMA caps with 64 active electrodes were used to record EEG signals in an acoustic and magnetic shield room. EEG signals were recorded at a sampling rate of 1200 Hz. A bandpass filter between 0.1 to 100 Hz and a notch filter of 50 Hz was applied to the raw EEG signals. The electrodes on the left earlobe and forehead were chosen as the reference and ground, respectively. The impedances for all the electrodes were maintained below 20 kΩ as recommended by the manufacturer. A Gazepoint GP3 eye tracker was set up to track eye movement during the BCI task. The sampling rate of the eye tracker was 60 Hz. A chin rest was used to fixate subjects’ head position (see Figure 1B). The data from eye tracker were recorded and synchronized with BCI2000 key events through a customized MATLAB script.

### Experimental Design and Protocol

Each subject was required to sit on a comfortable chair facing the center of a 24.5-inch LCD monitor. Before the start of each experimental session, native nine-point calibration of the eye tracker was implemented. The visual cues and feedback were displayed on the monitor. The distance between a subject and the monitor was set approximately 80 cm. Each of the participants took one session of an online BCI control task. Each session consisted of 10 runs of task blocks (around 5 min for each run), and each run included 30 trials of cursor control tasks. The task was a typical left vs. right cursor control task, but we required the subjects to control and fixate their gaze at a particular position during the mission. Before starting an experiment, each subject was allowed to practice one run of the experiment to be familiar with the tasks.

The trial structure was used in our previous research and was shown in Figure 1A (Meng et al., 2016; Meng and He, 2019). Each trial started with a blank screen lasting for 2 s, which was also used as the inter-trial interval. At the end of the blank screen, a yellow square serving as a target cue appeared either on the screen’s left side or right side, correspondingly a gray bar serving as the incorrect target appeared on the opposite side of the target cue. At the same time, a white cross appeared at the center of the screen with a left, right, or none arrow overlaying on the cross’s horizontal bar. The yellow target cue was displayed for two and half seconds in order to indicate the subject be prepared for the primary motor imagination task. While a white cross was used to instruct the secondary task, the center cross with or without an arrow was used to indicate where the subjects should orient and fixate their gaze (see Figure 1C). Subjects were asked to shift their gaze quickly toward the position according to the indicated cross arrow (left arrow: the center of a left target; right arrow: the center of a right target; a cross of none arrow: the center of the cross) and fixated their gaze during the entire trial. Then they performed motor imagination using repetitive movement imagination of their left hand or right hand corresponding to the left target or right target cue. At the end of the cue period, a round pinky cursor appeared in the center of the screen overlaying on top of the white cross. Subjects controlled the cursor moving toward the left or right target until it hit the correct (hit trial) or incorrect target (miss trial), or running out of 6 s without hitting the correct or incorrect target (abort trial). Then the cursor was frozen for 1 s to inform subjects of the current result. Note that, in many typical BCI studies, the BCI system would classify the trial to be either a hit trial or a miss trial. But in this study since there was a requirement of moving distance, those trials, in which subjects failed to reach the required distance, would be classified as abort trials. All subjects were instructed to perform the kinesthetic motor imagination from a first person’s perspective (Neuper et al., 2005).

**FIGURE 2:**
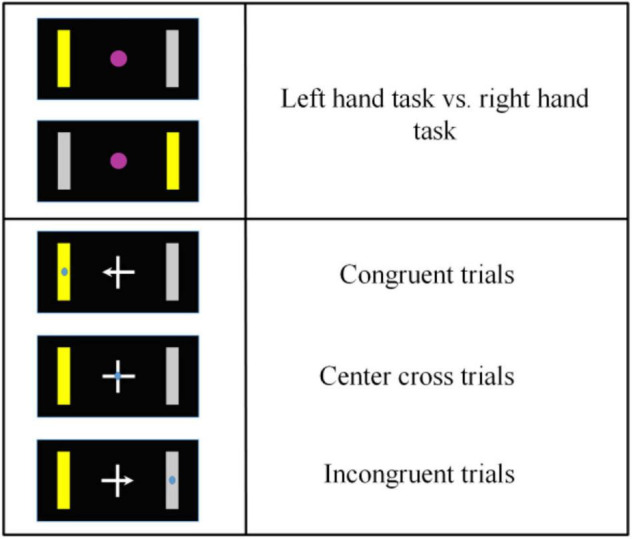
Overview of motor imagery tasks and gaze shift and fixation tasks. According to the congruence between a motor imagery task and a gaze shift and fixation position, there were three different control conditions, i.e., congruent trials, center cross trials and incongruent trials.

The target cues and the arrow of a center cross in each run were both assigned in a block randomized way. Thus, the number of left-hand and right-hand target cues, the number of arrowed crosses could be balanced accurately. Since the target center and the gaze center might be overlaid or at different locations, it resulted in three different BCI control conditions (see Figure 2): the target center and the gaze center were overlaid (a condition of congruent trials), the gaze center was on the cross center, but the target was on the peripheral side (a condition of center cross), the gaze center was on the opposite side of the target (a condition of incongruent trials).

### Online Signal Processing and Performance Evaluation

**FIGURE 3:**
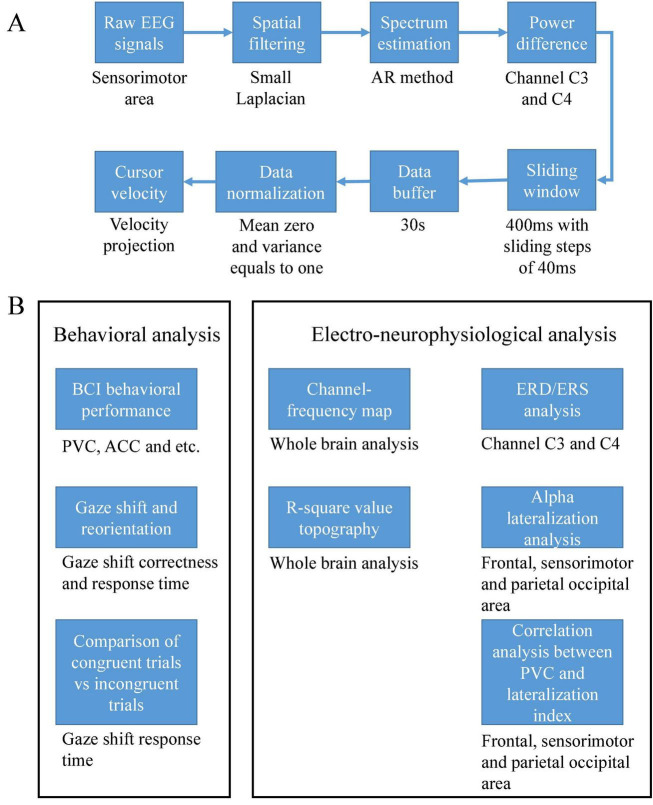
**(A)** Online signal processing flow chart and **(B)** schematic of the offline data analysis.

A similar setting of online signal processing as our previous work (Meng and He, 2019) was used in this study. The high alpha (10–14 Hz) power difference of channels C3 and C4 was used to control cursor movement. First, a small Laplacian filter (McFarland et al., 1997) was used to remove the surrounding common noise of channels C3 and C4. The power spectra of these two channels were estimated using an autoregressive (AR) method (McFarland and Wolpaw, 2008). A sliding window of 400ms was used to update the power spectrum continuously in a stepsize of 40 ms in order to increase the computation efficiency. Second, a single value of power difference between two channels was stored in a buffer of 30 s length, then the mean and standard deviation was calculated based on the buffered data. Thus, the output was a normalized value of the power difference using the above mean and standard deviation. Finally, the output signal was projected into the velocity of a cursor movement. The center cross was simultaneously programmed into the control sequence of a BCI2000 cursor task module (Schalk et al., 2004). The entire online signal processing procedures were illustrated in Figure 3A. Note that a certain time period was used to train the normalizer since input data has to be accumulated into the buffer. Thus the cursor did not move for the first trial of a BCI session. Subjects were still instructed to shift their gaze toward the prompt position and start their motor imagination after they perceived the target cue even if there was no feedback of cursor movement during the period of the first trial. Specifically, the first static trial was because the buffer needed to accumulate data to output the cursor’s movement.

Percent valid correct (PVC) (Meng et al., 2016; Meng and He, 2019) was used as an online performance measure to evaluate the BCI behavioral performance. PVC was calculated by the number of hits during each run divided by the total number of hits and misses. The number of abort trials was not taken into account in this metric. Since the number of abort trials was not considered in PVC, we also calculated the accuracy (ACC) defined as the number of hits divided by the total number of trials in each run. Since the cursor was controlled in a velocity-based fashion, the average duration and trajectory length of hit trials both represented extra measures that evaluated the efficiency of a BCI control. The mean duration and trajectory length of hit trials in each run were then averaged over all the runs of a session for each subject. The grand average duration and trajectory length of hit trials over subjects were investigated for each BCI control condition, respectively. Overall, the BCI behavioral performance including the PVC, ACC, feedback duration and the trajectory length were investigated. Several measures were used instead of a single ACC value in this study since these measures would give a comprehensive picture of subjects’ behavioral performance by BCI control.

### Calculation of Response Time of Gaze Shift and Reorientation

Besides the BCI behavioral performance, there was another behavioral performance of gaze shift and reorientation. The response time of an individual’s gaze shift and reorientation could be obtained using the following approach. At the beginning of each trial, a target cue appeared and lasted two and a half seconds. Gaze trajectory in the target cue period was captured and analyzed for each trial and each subject. However, the gaze trajectory could be noisy due to a small movement of the gaze. Here we thus set a threshold to the horizontal values of gaze positions, rounding the values toward three categorical numbers (0, left side; 0.5 center cross; 1, right side) by the following equation (1).


(1) Gaze_Xadjusted=0.5*round((X-0.5)*3)+0.5

X was the raw horizontal value of gaze position; X’s value ranged from 0 to 1. It was a serial number representing the position to the proportion of the screen width. Gaze_Xadjusted was the projected value after thresholding. Then a response time could be obtained at the rising or falling edge of the adjusted curve. Note that we used the time at the last rising or falling edge as a response time when the gaze shift and reorientation were correct. In some trials subjects might shift their gaze toward the incorrect target at the beginning, then they might realize their error and shift back to the correct target. In order to capture the response time of these trials, we decided to use the last rising or falling edge. The response time was invalid if the gaze shift and reorientation were wrong throughout the trial. The schematic of the offline data analysis was illustrated in Figure 3B.

### Calculation of R-square Value and Event-Related De-synchronization/Synchronization

R-square (*r*^2^) value, commonly used in BCI studies (Wolpaw and McFarland, 2004; Ramos-Murguialday et al., 2012; Nakanishi et al., 2017), was used to quantify the strength of each electrode to discriminate the left vs. right-hand imagination task. The r-square value was calculated at each electrode by the following equation (2):


(2) $$r^{2}=\frac{cov{\left(x,y\right)}^{2}}{var\left(x\right)var\left(y\right)}$$

The r-square value is the squared correlation coefficient for a single bivariate distribution computed from two sets of univariate data. Variable x is the measurement of condition one or condition two, y is the assigned value corresponding to condition one or condition two, e.g., y = +1 is assigned for condition one and *y* = −1 for condition two. Here the measurement of variable x is the power spectrum estimated for a particular channel in a specified frequency. Condition one corresponded to the right-hand imagination task, while condition two corresponded to the left-hand imagination task.

Then a topography of r-square value was generated to show how strongly the electrodes correlate with the discrimination of imagination tasks. In the offline analysis, the r-square values were calculated based on all of the trials including hit, miss, and abort trials in the frequency band of mu rhythm used for online control. The r-squared values were calculated for each subject and each session, then the grand average r-squared values and its topography were illustrated to show the strength of discrimination for each of the three BCI control conditions. Then event-related de-synchronization/synchronization (ERD/ERS) was calculated to characterize the dynamic change of band power activities relative to a baseline period in the electrodes used for online control (Graimann et al., 2002).

Additionally, r-squared values of each subject were contrasted between different control conditions, i.e., congruent trials vs. incongruent trials, congruent trials vs. center cross trials, and incongruent trials vs. center cross trials. Based on the contrasted r-squared values, brain electrodes/regions which differentiated different control conditions might be found. We then calculated the ERD/ERS dynamic processes in these electrodes, which emerged in the above contrast analysis. The functional hypothesis of ERD was suggested as a signature of task-relevant active cortical activities, and the ERS represented an inactive or inhibited cortical network (Pfurtscheller and Neuper, 2006). Thus, these metrics might be correlated with the task-relevant/inhibited cortical network to a certain degree. For each subject, the last 1.5 s of the inter-trial interval was selected as a baseline period. The time course of ERD/ERS was calculated from the beginning of the inter-trial interval to the 3 s after the feedback began. Here, 3 s were chosen since the average feedback duration was a little above 3 s. All of the trials were used to calculate ERD/ERS in a session, and a grand average over subjects was obtained for each BCI control condition, i.e., congruent trials, incongruent trials, and center cross trials.

Besides the commonly used ERD/ERS, the task-independent ERD/ERS lateralization index (Van Ede et al., 2011; Shu et al., 2018), which measured the average difference of each task between contra- and ipsilateral ERD, was calculated as well. In general, all of the electrodes that identified in the contrast analysis of the R square values were clustered in the left hemisphere or the right hemisphere. Then for each motor imagination task, the contralateral ERD of the identified electrodes in the corresponding hemisphere was first averaged spatially and then was substracted to its counterpart of average ipsilateral ERD. Last, the values for both of the motor imagination tasks were averaged and a task-independent ERD/ERS lateralization index was obtained. This metric was shown to be a sensitive metric to discover the neurophysiological change in both healthy populations and patients (Van Ede et al., 2011; Shu et al., 2018). Finally, the task-independent ERD/ERS lateralization index was contrasted between different BCI control conditions.

### Statistical Analysis

Statistical analysis was performed in Matlab R2019a and RStudio. The experiment adopted a randomized complete block design (Montgomery, 2017). Subjects were the block factors; The number of trials per condition was kept the same, i.e., 10 out of 30 trials per condition, and the order of each condition was kept relatively the same as well due to the randomization procedure. Therefore, within each subject, the conditions were as homogeneous as possible so that the treatment conditions could be compared under relatively homogeneous conditions. Factors such as fatigue, familiarity to the task would not affect each condition differently.

Since we have a limited number of subjects, a nonparametric approach of the Kruskal-Wallis test was used to evaluate the effect of three BCI control conditions on BCI behavioral performance, including the independent variables of PVC, ACC, feedback duration, and trajectory length. If the Kruskal-Wallis test was significant, a *post-hoc* analysis would be performed to determine which groups differ from each other group. Similarly, Wilcoxon’s sign rank test was used to compare the response time of gaze shift and reorientation between congruent trials and incongruent trials (the response time of the center cross trials was always zero if the subjects responded correctly, thus this response time was not considered).

An electrode-wise analysis of r-square values was performed and compared among different BCI control conditions. We performed a 3(three BCI control conditions) × 63(channels) repeated measure ANOVA to determine whether the r-square values in each BCI control condition and in each channel were significantly different. If the 3 × 63 repeated measure ANOVA test was significant in the main factors or the interaction, a *post-hoc* analysis would be performed to determine which main factors have a significant difference among levels. Specifically, if the main factor of BCI control conditions was significantly different, paired *t*-test with Bonferroni correction would be performed. On the other hand, if the main factor of channels was significant, paired *t*-test with false discovery rate (FDR) correction would be performed since we have 63 channels to compare. If the interaction between main factors was significant, it might indicate the r-square values of the channels differ from each other depending on the BCI control conditions. Therefore, contrast analyses of r-square values between paired control conditions would be performed, resulting in three paired comparisons, and the false discovery rate (FDR) was used for multiple comparison corrections.

Based on the analysis results of the r-square values, brain areas having a significant correlation with the motor imagination tasks could be identified. Then, brain region-wise analysis of lateralization process could be performed. Specifically, an N brain regions (identified in the analysis of r-square values) × 3(three BCI control conditions) × 15(15 time points uniformly distributed across 0 to 7.5 s) repeated measure ANOVA would be performed to determine whether the lateralization processes in each brain region and in each BCI control condition were significantly different. Similarly, if any main factor was significantly different, paired *t*-test with Bonferroni correction would be performed. Otherwise, if the interaction between main factors was significant, it might indicate the lateralization processes differ in each brain region depending on the BCI control conditions. Therefore, contrast analyses of the lateralization process between paired BCI control conditions in each brain area would be performed, resulting in three paired comparisons. The cluster-based permutation test was used to identify the significant clusters of periods of the lateralization process between paired BCI control conditions.

## Results

### Brain-Computer Interface Behavioral Performance

**FIGURE 4:**
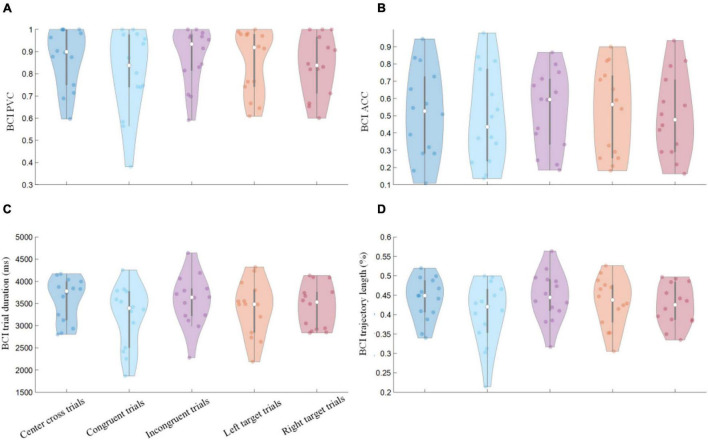
BCI behavioral performances of separate control conditions. Group average results of **(A)** PVC, **(B)** ACC, **(C)** feedback duration of single trials, **(D)** trajectory length of single trials. The labels of *x*-axis for all of the four figures were the same, representing the categorization of trials in terms of the motor imagery tasks (left vs. right) and the congruency between the motor imagery task and the gaze fixation task. Violin plots: shaded areas represent kernel density estimate of the data, white circles represent the median, and gray bars represent the interquartile range.

Percent valid correct and ACC defined in the methods were calculated separately for three BCI conditions, i.e., congruent, incongruent, and center cross trials. The individual and group average violin plots were shown in Figures 4A,B. Additionally, the PVC and ACC with respect to the left side (stimulus-Left) and the right side (stimulus-Right) target were investigated as well; the corresponding results were illustrated in Figure 4. The average PVCs ± standard error of the mean (SEM) for congruent trials, incongruent trials, and center cross trials were 80.59 ± 5.13, 87.44 ± 3.51, 87.48 ± 3.61%, respectively. The average ACCs ± SEM for congruent trials, incongruent trials, and center cross trials were 49.35 ± 7.37, 53.09 ± 6.10, 51.24 ± 6.99%, respectively. The Kruskal-Wallis test was performed between paired control conditions, resulting in three paired comparisons (center cross vs. congruent, center cross vs. incongruent, and congruent vs. incongruent). Bonferroni’s *post-hoc* test was used to correct for multiple comparisons. The statistical analysis revealed that there was no significant difference in behavioral performance between different control conditions. Furthermore, left-target and right-target trials were analyzed separately to see whether there was any difference between them. The average PVCs ± SEM for left-target and right target trials were 85.18 ± 3.89, 83.86 ± 3.65%, respectively. The average ACCs ± SEM for left-target and right target trials were 52.08 ± 6.93, 50.48 ± 6.00%, respectively. There was no significant difference between left-target and right-target trials in PVC and ACC.

Individual and group average violin plots of hit trials’ duration and trajectory length were shown in Figures 4C,D. The group average durations of three BCI control conditions were 3.21 ± 0.19, 3.56 ± 0.15, 3.59 ± 0.13 s, respectively. The group average trajectory lengths of three BCI control conditions were 0.40 ± 0.02, 0.45 ± 0.02, 0.44 ± 0.02 units of the screen width, respectively. The statistical analysis revealed that there was no significant difference in behavioral performance between different control conditions. The average durations ± SEM for left-target and right target trials were 3.39. ± 0.16, 3.43 ± 0.13 s, respectively. The average trajectory lengths ± SEM for left-target and right target trials were 0.43 ± 0.02, 0.43 ± 0.01%, respectively. There was no significant difference between left-target and right-target trials in feedback duration and trajectory length.

### Behavioral Performance of Gaze Shift and Reorientation

**FIGURE 5:**
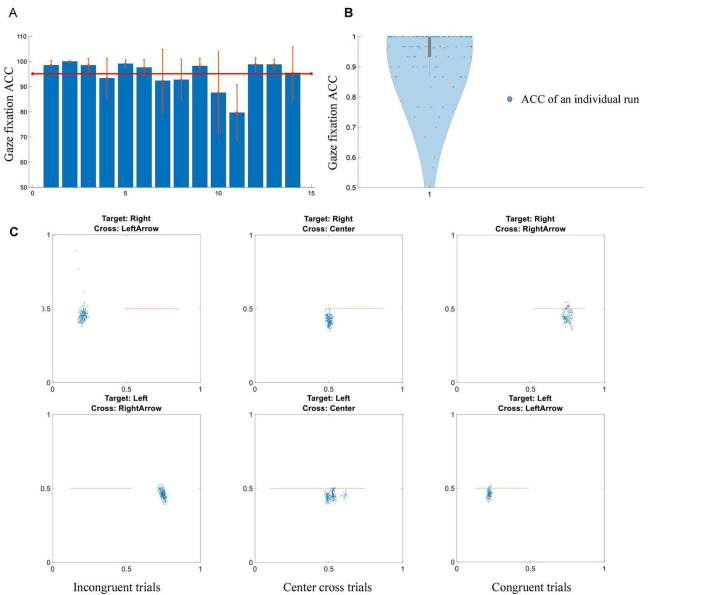
Gaze shift and fixation performance. **(A)** Individual accuracy ± standard error of gaze shift and fixation. The red line represents a mean value of fourteen subjects. **(B)** Violin plot of all of subjects’ performance in terms of individual runs. **(C)** An example of gaze fixation for a particular subject in a randomly selected run. Blue dots represent gaze fixation points, orange lines demonstrate the trajectories of a cursor’s movement. Left column: incongruent trials, middle column: center cross trials, right column: congruent trials.

Subjects’ behavioral performance of gaze orientation and fixation was analyzed and displayed in Figure 5. Overall, the group average accuracy is 95.1 ± 2.4%, which means the majority of subjects could complete the gaze orientation and fixation with very high accuracy. The chance level of gaze orientation and fixation was 33.33%. Each subject’s behavioral performance of gaze orientation and fixation in individual runs was shown in the violin plot of Figure 5B. The horizontal and vertical positions of a particular subject’s gaze endpoint during the feedback period were shown in the scatter plot of Figure 5C. An orange line represented a cursor’s trajectory in the feedback period. Representative examples of congruent trial, incongruent trial and center cross trials were depicted in the left, middle and right columns of Figure 5C. The subject had a 100% accuracy of gaze shift and reorientation for this particular run.

**FIGURE 6:**
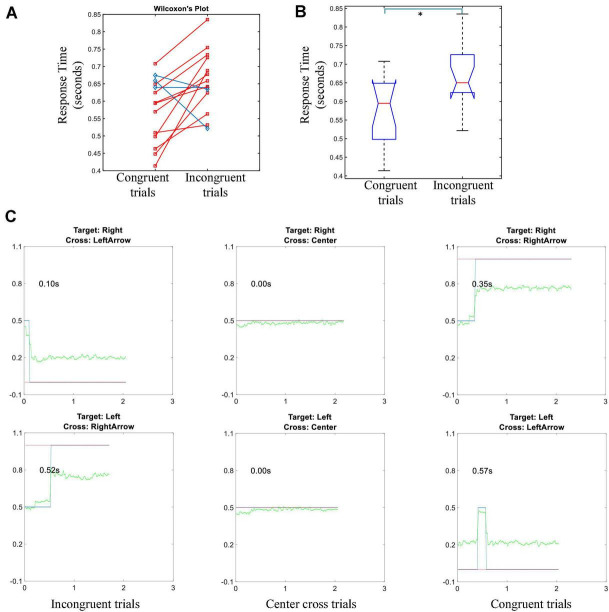
Response time of the gaze shift and fixation task. **(A)** The average response time of congruent trials vs. incongruent trials for each individual, the distribution is used for Wilcoxon’s sign rank test. Red lines represent the response time of incongruent trials is longer than that of congruent trials. Blue lines show that the opposite holds. **(B)** Box plot of the group average response time of congruent trials vs. incongruent trials (* means *p* < 0.05). **(C)** An example of thresholding the gaze shift and fixation for a particular subject in a randomly selected run. Green lines represent gaze fixation trajectory in the pre-feedback period; blue lines demonstrate the rectified trajectories after thresholding; red lines indicate the correct value for an indicated gaze fixation cue. Left column: incongruent trials, middle column: center cross trials, right column: congruent trials.

The individual’s average response time of gaze shift and reorientation was shown in Figure 6A, their grand average was shown in Figure 6B. Due to the gaze position was in the center cross at the beginning of each trial, only response times of congruent and incongruent conditions were available. A red line in Figure 6A meant the response time of an incongruent trial was longer than the response time of a congruent trial for the subject, while a blue line meant the opposite result held. Eleven of the fourteen subjects showed an increase in response time for the incongruent trials. The average response times of congruent and incongruent trials were 0.57 ± 0.09 and 0.65 ± 0.09 s, respectively. Wilcoxon’s signed-rank test showed a significant difference between the response times of congruent and incongruent trials. This difference indicated that the majority of subjects took a longer response time to shift and reorient their gaze to the correct target in the incongruent trials, but there were differences among subjects.

### Brain-Computer Interface R-square Value Analysis in Three Conditions

**FIGURE 7:**
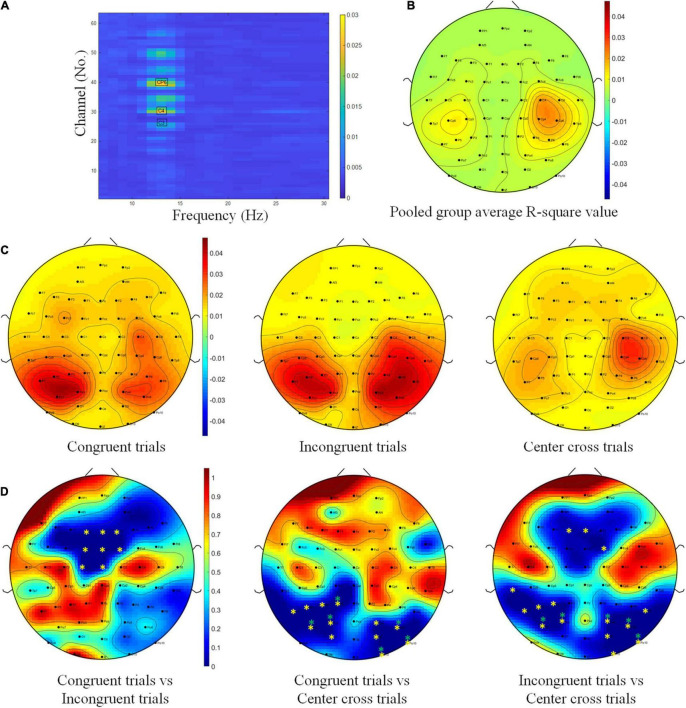
R-square value topography maps and statistics. **(A)** Channel-frequency map of group average R-square values over fourteen subjects. **(B)** Topography of the pooled group average R-square values over fourteen subjects. **(C)** Topographies of group average R-square values over fourteen subjects with respect to congruent trials, incongruent trials and center cross trials. The color bar for all three topographies of this row was the same in order to have a fair comparison. **(D)** Contrast analysis of R-square values between congruent trials and incongruent trials, between congruent trials and center cross trials, between incongruent trials and center cross trials. The color bar for all three *p*-value maps was the same (* means *p* < 0.05, ^**^ means *p* < 0.01).

First, the electrode-wise r-square values were calculated and compared among different BCI control conditions. As we planned in the statistical analysis, a 3(three BCI control conditions) × 63(channels) repeated measure ANOVA was conducted to determine whether the r-square values in each channel and in each BCI control condition were significantly different. BCI control conditions did not show significant effect on r-square values, *F*(2,26) = 2.79, *p* = 0.08, η_*ges*_^2^ = 0.02. However, channels had a significant effect on r-square values, *F*(62, 806) = 3.84, *p* < 0.001, η_*ges*_^2^ = 0.15. Furthermore, the interaction between BCI control conditions and channels was significant, *F*(124,1612) = 3.55, *p* < 0.001, η_*ges*_^2^ = 0.07, which indicated that channels located in different brain regions might have a significant effect on r-square values, but it depended on the BCI control condition. Due to we did not find any significant difference for the main factor of BCI control conditions, all of the trials were pooled together to get a channel frequency map of R-square values regardless of their condition, the result was plotted in Figure 7A. This map indicated that channels C3, C4, and channel CP6, in the high alpha frequency band (10–14 Hz), were most important to discriminate the left and right motor imagery task. The topography of R-square shown in Figure 7B also proved that channels C3, C4, and channel CP6 were with higher R-square value. This was consistent with the prior knowledge of hand-related motor imagery tasks.

Since a significant interaction effect between BCI control conditions and channels was found, we would like to visually explore the group average R-square topography for each BCI control condition first. Based on this information, grand average topographies of R-square values were calculated and plotted for congruent trials, incongruent trials, and center cross trials, respectively, in Figure 7C. The topography of R-square values for the center cross condition, the typical condition in the conventional experiment, showed a concentration of activities around the C3, Cp5, C4, and Cp4. This concentration around the sensorimotor area was consistent with many previous studies (Wolpaw and McFarland, 2004; Meng et al., 2016; Meng and He, 2019). The topographies of R-square values for the congruent and incongruent trials, however, showed a shift toward the posterior parietal area.

Further contrast analysis was performed between each pair of conditions because the overall ANOVA analysis indicated the impact of channels depending on the BCI control condition. Contrast analysis of R-square values between each pair of conditions were shown in Figure 7D. Since we have 63 channels in total, Bonferroni correction would be too conservative to find any significant channels, FDR was used for correction of multiple comparisons. The permutation test with FDR correction for multiple comparisons showed significant differences in R-square values in the frontal area, including the channels F1, Fz, F2, Fc1, Fcz, Fc2, C1, and Cz when comparing the congruent trials with incongruent trials. In addition, the significant differences of R-square values were displayed in the parietal occipital area, including P7, P5, P3, P1, Po7, Po3, O1, Po4, Po8, O2, O10, and Po10 when comparing the congruent trials with the center cross trials. The results of contrast analysis for incongruent trials vs. the center cross trials highlighted both the frontal area and the parietal occipital area, including the frontal channels F1, Fz, Fc2, and the parietal occipital channels Tp7, P7, P5, P3, Po7, Po3, Po4, P8, Po8, O2, O10, and Po10.

### Event-Related De-synchronization/Synchronization Modulation Analysis in Three Conditions

**FIGURE 8:**
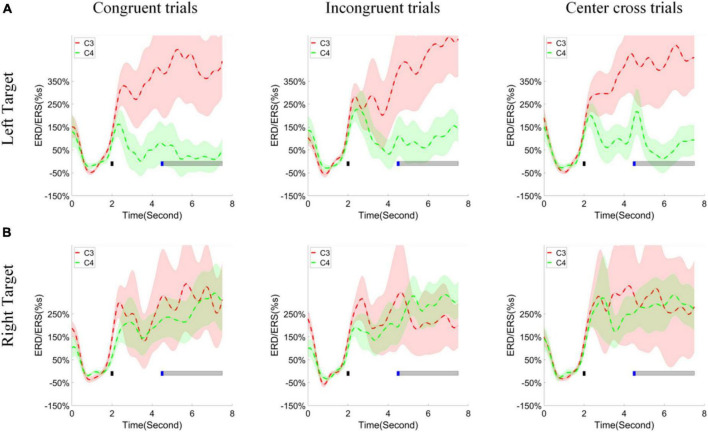
ERD/ERS values and statistics. **(A)** The group average of time varying ERD/ERS values for the left hand task (the upper row) and **(B)** right hand task (the bottom row) over fourteen subjects. The columns separated the results for congruent trials, incongruent trials and center cross trials, respectively. The target cue appeared at the end of second 2, which was indicated by a short black bar. The cursor feedback began at the second of 4.5, which was marked by a short blue bar. The light gray bar represented the feedback period.

According to the pooled group average topographies of R-square values, channels C3 and C4 played significant roles in discriminating the left and right-hand tasks. Thus, the change of ERD/ERS values of high alpha band across time over the channels C3, C4 was calculated and illustrated in Figure 8 for the left target and right target (upper and lower row), congruent trials, incongruent trials, and center cross trials (left, middle and right column), respectively. The channel C3 exhibited significant ERS during the feedback period of the left target’s cursor control, which was marked by a grayed horizontal bar, regardless of control conditions. The small blue square indicated the starting time point of a feedback period. On the other hand, channel C4 showed less clear ERD or ERS compared to the baseline for the left target’s cursor control. From the figure, we could see that a separation of ERD/ERS activities between channel C3 and C4 happened at a few hundreds of milliseconds after the target cue appeared. However, both channels C3 and C4 showed inseparable ERS during the feedback period of the right target’s cursor control.

According to the contrast analysis of R-square values between paired BCI control conditions, frontal and parietal occipital areas may play roles in the current experimental design. Therefore, the task-independent ERD/ERS lateralization index was calculated for frontal, sensorimotor, and parietal occipital regions separately. We performed a 3(three BCI control conditions) × 15(15 time points uniformly distributed across 0s to 7.5 s) × 3 (brain regions identified in the analysis of r-square values) repeated measure ANOVA as planned in the statistical analysis. The main factor of BCI control conditions did not show significant effect on the alpha ERD/ERS lateralization indexes, *F*(2,26) = 3.19, *p* = 0.06, η_*ges*_^2^ = 0.03. Similarly, neither brain regions show significant effect on the alpha ERD/ERS lateralization indexes, *F*(2,26) = 0.95, *p* = 0.40, η_*ges*_^2^ = 0.01. However, time did show significant effect on the alpha ERD/ERS lateralization indexes, *F*(14,182) = 6.29, *p* < 0.001, η_*ges*_^2^ = 0.03. Furthermore, the interaction between BCI control conditions and time [*F*(28,364) = 3.92, *p* < 0.001, η_*ges*_^2^ = 0.06], the interaction between BCI control conditions and brain regions [*F*(4,52) = 5.78, *p* < 0.001, η_*ges*_^2^ = 0.06] and the interaction among BCI control conditions, time and brain regions [*F*(56,728) = 2.89, *p* < 0.001, η_*ges*_^2^ = 0.06] were all significant. This might indicate the alpha ERD/ERS lateralization process might have significant differences in different periods of time, but the difference depended on the BCI control conditions and brain regions.

**FIGURE 9:**
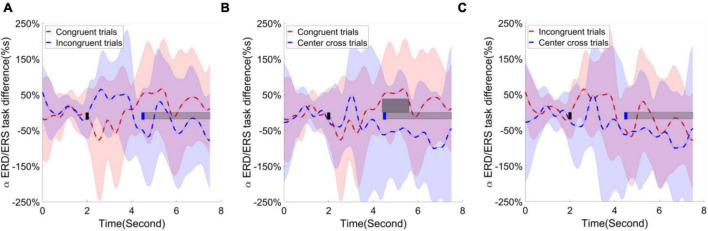
Group average alpha ERD/ERS task difference across the time in the frontal area. Shaded areas represented the stand error of an alpha ERD/ERS task difference. The target cue appeared at the end of second 2, which was indicated by a short black bar. The cursor feedback began at the second of 4.5, which was marked by a short blue bar. The light gray bar represented the feedback period. The dark gray bar demonstrated the period of significant difference. Contrast analysis of alpha ERD/ERS task difference process in the frontal area between **(A)** congruent trials and incongruent trials, **(B)** between congruent trial and center cross trials, **(C)** between incongruent trials and center cross trials.

**FIGURE 10:**
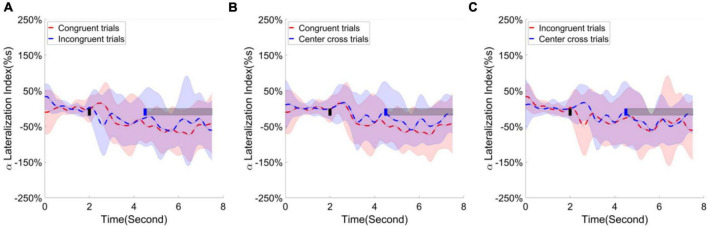
Group average alpha lateralization index across the time in the sensorimotor area. Shaded areas represented the stand error of a lateralization index. The target cue appeared at the end of second 2, which was indicated by a short black bar. The cursor feedback began at the second of 4.5, which was marked by a short blue bar. The light gray bar represented the feedback period. Contrast analysis of lateralization process in the sensorimotor area **(A)** between congruent trials and incongruent trials, **(B)** between congruent trial and center cross trials, **(C)** between incongruent trials and center cross trials.

Following the statistical analysis results, first, the alpha ERD/ERS lateralization index, i.e., the difference of average ERD/ERS activities over the channels F1, Fz, F2, Fc1, Fcz, Fc2, C1, Cz between right-hand trials and left-hand trials, were calculated and plotted over the frontal area in Figure 9. The cluster-based permutation test identified one significant cluster (*p* < 0.05), extending more than 1 s after the feedback begins when comparing the congruence trials with the center cross trials. Second, the alpha ERD/ERS lateralization index over the sensorimotor cortex was calculated and illustrated in Figure 10. It measured an average difference between contralateral and ipsilateral ERD/ERS activities, which was not affected by a task type (left-hand or right-hand trials). The contrast of average ERD/ERS activities over the left cortex (Fc3, C5, C3, C1, and Cp3) between right-hand trials and left-hand trials, and the contrast of average ERD/ERS activities over the right cortex (Fc4, C6, C4, C2, and Cp4) between left-hand trials and right-hand trials were averaged to get a single value of alpha ERD/ERS lateralization index. The cluster-based permutation test did not identify any significant cluster between any paired comparisons.

**FIGURE 11:**
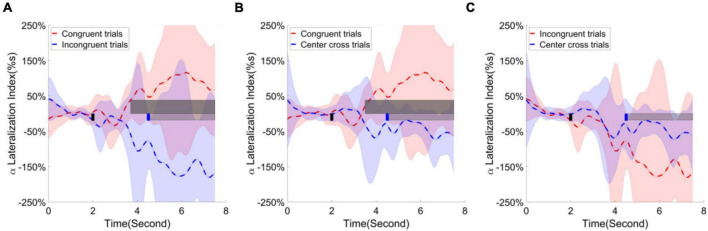
Group average alpha lateralization index across the time in the parietal occipital area. Shaded areas represented the stand error of a lateralization index. The target cue appeared at the end of second 2, which was indicated by a short black bar. The cursor feedback began at the second of 4.5, which was marked by a short blue bar. The light gray bar represented the feedback period. The dark gray bar demonstrated the period of significant difference. Contrast analysis of lateralization process in the parietal occipital area between **(A)** congruent trials and incongruent trials, **(B)** between congruent trial and center cross trials, **(C)** between incongruent trials and center cross trials.

Finally, the alpha ERD/ERS lateralization index over the parietal occipital cortex was calculated and illustrated in Figure 11. The contrast of average ERD/ERS activities over the left parietal occipital cortex (P5, P3, Po3, Po7, the most significant channels in contrast analyses of r-square values) between right-hand trials and left-hand trials, and the contrast of average ERD/ERS activities over the right cortex (Po4, Po8, O2, Po10, the most significant channels in contrast analyses of r-square values) between left-hand trials and right-hand trials were averaged to get a single value of alpha ERD/ERS lateralization index. The cluster-based permutation test identified two significant clusters; one is for comparing congruent trials and incongruent trials, the other is for comparing congruent trials and center cross trials. The substantial separation in lateralization index began at about 1 s before the feedback cursor appeared and lasted for the entire feedback period we analyzed. This significant separation showed in the comparisons between congruent and incongruent trials and between congruent trials and center cross trials.

### Correlation Analysis of Lateralization Index in Three Brain Areas

**FIGURE 12:**
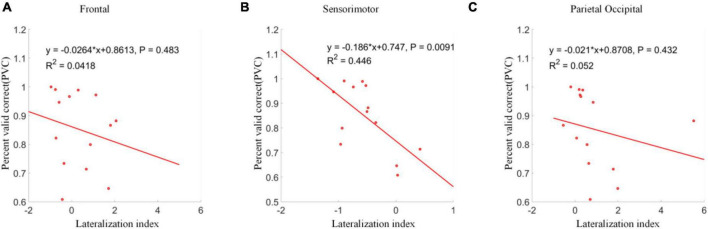
Linear regression analyses between lateralization indexes and PVCs. **(A)** Frontal area. **(B)** Sensorimotor area. **(C)** Parietal occipital area. A significant linear correlation was only shown between lateralization indexes and PVCs in sensorimotor area.

The correlations between lateralization indexes over frontal, sensorimotor and parietal occipital areas and PVC were analyzed separately. The results were shown in Figure 12. Although significant clusters were found between different conditions in frontal and parietal occipital regions, no significant correlation between lateralization indexes over frontal and parietal occipital areas and PVC was found. However, a significant linear correlation between lateralization index over the sensorimotor area and PVC was found (*p* < 0.01). A more negative lateralization index corresponded to a higher online classification accuracy in terms of PVC.

## Discussion

Previously, gaze orientations were not controlled rigorously; subjects might use different strategies to set up their gaze orientations in the experiment (Wolpaw et al., 2002; Wolpaw and McFarland, 2004; Meng et al., 2016; Meng and He, 2019; He et al., 2020). We set out to investigate whether the BCI behavioral performances were various under different gaze orientations. Thus, three conditions of gaze orientations and fixation were designed in the current study. Subjects were required to perform two tasks simultaneously at the appearance of the target cues for both motor imagery and gaze reorientation. Their behavioral gaze shift and reorientation performance showed a high completion rate and accuracy (see Figure 5). This high completion rate meant that subjects could follow the instructions successfully. It excluded the trivial possibility that the non-significant difference between different BCI control conditions was due to the inability to complete the dual task under any BCI control condition.

Furthermore, the BCI behavioral performances did not show any significant difference in terms of PVC, ACC, time duration of control, and trajectory length. This might indicate that the gaze orientation or positions might not significantly affect the BCI behavioral performance, no matter whether it was congruent with the target position or not. Thus, the results were consistent with the convention that motor imagery-based BCI was a gaze-independent system.

However, the response time of gaze shift and reorientation for congruent trials was significantly shorter than for incongruent trials (see Figure 6). This response time result was reasonable since the subjects might need a longer time to process the inconsistent information and make a correct judgment to follow the instruction. This was also corroborated by the contrast analysis between congruent trials and incongruent trials, between incongruent trials and center cross trials. The significant difference of alpha R-square values over the frontal areas indicated that frontal regions might play a functional role in processing conflict information in the incongruent trials (Ehlis et al., 2005; Cohen and Ridderinkhof, 2013). Due to the experimental design, the subjects were required to fixate their gaze at the center of the cross; thus, the response time of gaze shift and reorientation for center cross trials was not available.

Notice that, we explored the possibility of performing a motor imagery (MI) task and an SSVEP task simultaneously in our previous work (Edelman et al., 2018). The results of this previous study showed that subjects could perform two typical BCI tasks without interfering each other significantly. The direct evidence was that the BCI performance of either single modality (MI or SSVEP) in multi-tasking (MI+SSVEP) did not decrease compared to its corresponding performance in a single task (MI only or SSVEP only) which required significantly less demanding mental effort. This previous work demonstrated a particular case of combining a motor imagery BCI with another SSVEP BCI modality. A direct hypothesis following the previous experiment was that gaze fixation might be a secondary task which did not interfere with the motor imagery task. Because most of the visual stimulus dependent BCI paradigm relied on the gaze fixation to the attended target, the results of this current study built the foundation to expand the multi-tasking modality.

The previous work in Edelman et al. (2018) found a significant difference between the congruent trials and incongruent trials (see Figure 6C in Edelman et al., 2018). However, the experimental results in this study did not show statistical significance among different conditions that was to say the results did not support the hypothesis of which there was a significant difference between the congruent trials and incongruent trials. The reason might be that the 1D task in this study was considerably easier than the 2D task in the previous study. There were not many visual resources conflicts between the gaze shift, reorientation and motor imagery.

The ERD/ERS process over the sensorimotor area was consistent with the previous studies (Meng et al., 2016; He et al., 2020). Especially, the ERS was pronounced in channel C3, and ERD/ERS was almost at a baseline level in channel C4 for the left target trials. However, ERS was substantial in both channels C3 and C4 for the right-hand target trials, but their difference was inseparable (see Figure 8). This ERD/ERS result was more or less consistent with our previous studies (Meng and He, 2019). The analysis of the ERD/ERS lateralization index showed a similar negative level regardless of the control conditions (see Figure 10). This demonstrated that contralateral alpha activities were smaller than the ipsilateral alpha activities over the sensorimotor area when using motor imagination as control strategies.

The contrast analysis between congruent and center cross trials, between incongruent trials and center cross trials showed that the posterior parietal occipital cortex was significantly activated. This activation of parietal occipital region could be interpreted that when subjects maintain their gaze at the indicated position, they also had to covertly pay attention to the movement of a cursor so that the cursor could successfully hit the correct target. Plenty of studies showed that covert attention would modulate the alpha rhythm of the posterior parietal occipital cortex (Worden et al., 2000; Kelly et al., 2006; Thut et al., 2006). Attention to location or motion direction both activated the parietal and occipital areas (Corbetta and Shulman, 2002). One of our previous studies also proved that it could serve as a supplementary control dimension to complete the 3D cursor control by combining the motor imagery and covert attention modulation (Meng et al., 2018).

Furthermore, the analysis of ERD/ERS lateralization over the posterior parietal occipital area showed a pronounced positive level for congruent trials, a pronounced negative level for incongruent trials. The contrast analysis between congruent and incongruent trials revealed a significant period starting around 1 s before the feedback cue appeared and lasting for the entire analyzed feedback period. This significant period indicated a strong lateralization effect happened for both congruent and incongruent trials. Additionally, early lateralization before cue appeared might suggest the covert attention was paid before the feedback cursor appeared. An increase of lateralization index might indicate the following hypothesis. Covert attention to moving objects might generate more significant lateralization after the cursor appeared than covert attention to static or nonspecific objects. However, we do not have experimental data to support this speculation.

Furthermore, lateralization directions of congruent trials and incongruent trials were the opposite. The opposite lateralization direction was caused by the calculation method. First, subjects fixated their gaze at the position opposite to an indicated target in the incongruent trials; thus, the cursor would move away from the gaze center if the motor imagination correctly controlled the cursor. Since the direction of covert attention was congruent with the target, the lateralization index should be negative due to contralateral ERD and ipsilateral ERS. On the contrary, subjects maintained their gaze at the center of an indicated target in the congruent trials; thus, the cursor would move toward the gaze center if the motor imagination correctly controlled the cursor. The lateralization index was positive due to contralateral ERD and ipsilateral since the direction of covert attention was incongruent with the target (i.e., for left target, covert attention is on the right side).

The correlation analysis of lateralization index with PVC from different brain areas only showed a significant linear correlation between the sensorimotor area and the PVC. Although parietal occipital area was significantly activated during the control, it had little effect on the accuracy of cursor control. This uncorrelated parietal occipital activity was reasonable since the cursor was supposed to be controlled by the motor imagination, which should activate the sensorimotor area. Thus, the neural activities in the parietal occipital area did not influence the BCI performance, even though covert attention strongly activated it.

### Limitations and Future Work

In this study, we included a group of subjects including a single female and a few subjects who had BCI experience previously. Previous studies showed that the gender (Cantillo-Negrete et al., 2014) and the subjects’ experience (Pillette et al., 2021) might be influential factors to the study results. First, because the gender and experience were both across subjects factors, it would not affect our investigating factors (within-subject factors) significantly. Second, a few subjects only got access to the BCI paradigm previously. All of the subjects were naïve to the dual tasks before participating in this study. To remove the potential influence of familiarity to the dual tasks, additionally, all of the subjects were given a practicing run to be familiar with the dual tasks. Last, we had used a random block design in our paradigm to further alleviate the potential influence of familiarity and fatigue since the sequence of the BCI control tasks might affect the BCI performance. Due to these reasons, the experienced subjects in this study were not excluded considering a limited number of subjects.

A user would more likely control a robotic arm or wheelchair to perform 2D tasks in real life. But the 2D experiment would be much more complex than the 1D experiment of this study. First, the number of congruent and incongruent trials would be quite different, this would require more trials, which were challenging, for each session. Second, there might be difference between the incongruent trials, which made the comparison among conditions less homogeneous than the current study. Thus, we did not conduct the 2D experiment in this study. But the 2D experiment would be an interesting exploration for the future work.

## Conclusion

In this study, motor imagination of left-hand vs. right-hand has demonstrated comparable BCI behavioral performance under three control conditions of gaze shift and fixation. A group of fourteen subjects’ PVC has shown a performance of over 80% accuracy. Further analyses of individuals’ response time reveal that subjects respond faster for congruent trials than incongruent trials. During the feedback control, covert attention to a cursor’s movement induces lateralized alpha activities over the parietal occipital area. This lateralization process displays a significant deviation of baseline level when comparing the congruent trials to the incongruent trials, and when comparing the congruent trials to the center cross trials. This lateralization process starts about 1 s before the feedback begins, which indicates that covert attention is paid to the cursor early than it moves. Nevertheless, neither gaze shift and fixation on different positions nor covert attention to a cursor’s movement affect the BCI behavioral performance. These independent brain activities might be advantageous to BCI real applications such as controlling a robotic arm and a wheelchair.

## Data Availability Statement

The raw data supporting the conclusions of this article will be made available by the authors under a material transfer agreement with Shanghai Jiao Tong University, without undue reservation.

## Ethics Statement

The studies involving human participants were reviewed and approved by The Institutional Review Board of Shanghai Jiao Tong University. The patients/participants provided their written informed consent to participate in this study.

## Author Contributions

JM wrote the first draft of the manuscript. ZW revised the draft of the manuscript. JM, ZW, SL, and XZ edited the manuscript. JM and XZ conceived and designed the experimental paradigm and wrote the manuscript. JM, ZW, and SL performed the research and analyzed the data. All authors contributed to the article and approved the submitted version.

## Conflict of Interest

The authors declare that the research was conducted in the absence of any commercial or financial relationships that could be construed as a potential conflict of interest. The handling editor declared a past collaboration with one of the authors JM.

## Publisher’s Note

All claims expressed in this article are solely those of the authors and do not necessarily represent those of their affiliated organizations, or those of the publisher, the editors and the reviewers. Any product that may be evaluated in this article, or claim that may be made by its manufacturer, is not guaranteed or endorsed by the publisher.

## Supplementary Material

Supplementary Video 1 The video demonstrated the first ten trials of a particular subject in a run. The first trial was an abort trial (center cross condition), following a miss trial in the second one (incongruent condition). Then the subject succeeded to hit all of the trials from trial three to trial six (trial three was the congruent condition). Trials seven and eight were both miss trials. Trials nine and ten were both hit trials.
